# Gestational hypertension and progression towards preeclampsia in Northern Ethiopia: prospective cohort study

**DOI:** 10.1186/s12884-021-03712-w

**Published:** 2021-03-30

**Authors:** Awol Yemane, Hale Teka, Sumeya Ahmed, Haftom Temesgen, Elizabeth Langen

**Affiliations:** 1 College of Health Sciences, Department of Obstetrics and Gynecology, Mekelle University, Ethiopia Witten Street, Mekelle, Ethiopia; 2 College of Health Sciences, Department of Health Systems, Mekelle University, Mekelle, Ethiopia; 3 College of Health Sciences, Department of Biostatics, Mekelle University, Mekelle, Ethiopia; 4 Department of Obstetrics and Gynecology, Division of Maternal Fetal Medicine, University of Michigan, Ann Arbor, USA

**Keywords:** Gestational hypertension, Preeclampsia, Progression, Low‐resource setting, Ethiopia

## Abstract

**Background:**

Preeclampsia (PE) is one of the main causes of medical complication of pregnancy and is the main cause of perinatal mortality and morbidity. It is one of the top causes of maternal mortality in Ethiopia. Also known as transient hypertension, gestational hypertension (GH) is increased blood pressure during pregnancy without proteinuria, which is expected to return to normal by the 12th-week postpartum visit. PE is GH with proteinuria and /or other systemic manifestations. Evidence from high income countries show that GH significantly progresses towards PE. To our knowledge, this is the first study on the progression of GH towards PE in an African setting. The objective of this study is, therefore, to assess the incidence of GH, progression towards PE and factors associated with progression in Ethiopia.

**Methods:**

This is a prospective cohort study conducted at Ayder Comprehensive Specialized Hospital (ACSH) and Mekelle General Hospital (MGH), the largest referral centers in Northern Ethiopia. Two hundred and forty women with GH were enrolled and followed up until delivery. Clinical and laboratory data at initial presentation and at follow-up were compared among women who progressed towards PE and who remained with the diagnosis of GH. Logistic regression analysis was employed to model the combined effects of the clinical and laboratory data as significant predictors of progression from GH to PE.

**Result:**

The incidence of GH in this study was 6 % (4.9–8.5). The rate of progression was 17.1 % (13.4–23.8). Previous history of GH, anemia during pregnancy, previous second-trimester spontaneous abortion were significant predictors of progression.

**Conclusions:**

There is a high rate of progression of GH towards PE. In a resource-limited setting where predictive and diagnostic tools are scarce, clinical profile of women should be taken into consideration for prediction and diagnosis of PE.

## Background

Globally hypertension is the most frequent medical complication of pregnancy, occurring in 10 % of pregnancies and includes a range of conditions, including chronic hypertension, gestational hypertension (GH), and preeclampsia (PE) [1]. In Ethiopia, the prevalence of all forms of hypertensive disorders of pregnancy varies from 1.8 to 10 % [2]. GH also known as transient hypertension is the new onset of hypertension after 20 weeks of gestation  [1]. GH is expected to return to normal by the 12th-week postpartum visit. PE, on the other hand, is defined as GH with proteinuria and/or systemic manifestations( headache, right upper quadrant pain, epigastric pain, blurring of vision, organ function derangement, low platelet count) and is associated with more grave maternal and neonatal outcomes [1, 3, 4].

In a 10 year study by Walker and colleagues in a Calgary health region in Canada, the incidence of GH was found to be 6.3 % [5]. In another study in Nigeria, the incidence of GH was 5.9 % [6]. Saudan et al. reported 15–25 % of women who are initially admitted with GH progress to PE [4].

Globally, 10–15 % of maternal deaths are directly related with PE and eclampsia [7]. Complications are a lot worse in resource-limited settings which are associated with a lack of antenatal care follow up and delays in diagnosis and treatment [2, 8]. Previous studies in Ethiopia show that perinatal mortality associated with hypertensive disorders of pregnancy is one of the highest in the world, 111/1000 live births [9]. In a retrospective hospital-based study in Ethiopia, PE and eclampsia accounted for 35 % of maternal deaths, highlighting the significance of this disorder in both maternal and fetal outcomes [8].

Although hypertensive disorders of pregnancy are more common in low and middle income countries than they are in the high income ones, little is known about the incidence of GH and progression towards PE [2, 4]. To our knowledge to date, no published accessible data is available on the incidence of GH and factors affecting its progression towards PE in this country. Women with GH may be managed safely as outpatients, and it would be helpful to know both the absolute risk of progression from GH to PE and the factors at the initial presentation which predict this progression [1]. The objective of this study is, therefore, to assess the incidence of GH, progression towards PE and factors associated with progression in Ethiopia.

## Methods

### Operational definitions

GH was defined as the onset of hypertension (systolic BP > = 140 mmHg and /or diastolic BP > = 90 mmHg) after 20 weeks of gestation [1]. Hypertension in these women was confirmed following repeated BP measurements during a 4-6-h day assessment unit visit or during admission.

PE was diagnosed when one or more of the following maternal systemic complications accompanied hypertension: proteinuria > = 2 + on dipstick urine analysis, renal impairment (plasma creatinine > = 1.2 mmol/L), hepatic dysfunction (aspartate transaminase > = 70 or double rise from baseline, right upper quadrant pain and/or sever epigastric pain, hematologic abnormality (platelet count < 100,000/L), neurologic features ( blurred vision, headache ,abnormal body movement ) or sever hypertension (systolic BP > = 160 mmHg and/or diastolic BP > = 110 mmHg ). Abnormal body movement is generalized tonic clonic type of seizure. Right upper quadrant pain is right sided upper abdominal pain, just beneath the ribs.

### Study setting

This is a prospective cohort study conducted in two public Hospitals of Mekelle City. Ayder Comprehensive Specialized Hospital (ACSH) and Mekelle General Hospital (MGH). Ayder Comprehensive Specialized Hospital, a tertiary care center for a catchment area over 8 million people in north Ethiopia. Mekelle General Hospital is the second largest Hospital in Tigrai region. High risk pregnant women including hypertensive disorders of pregnancy are referred to both hospitals for management.

Study period was between August 1, 2016 and July 31, 2017. Every case of GH seen during the study period at the hospitals were enrolled in the study Patients with previously known essential hypertension, renal disease or other secondary cause of hypertension were excluded.

### Clinical and laboratory data

The data was collected by 6 trained midwives and 4 trained resident physicians at both hospitals. The following clinical and laboratory data at initial presentation were recorded in women with GH : age, parity, abortion history, gestational age, educational status, family monthly income, marital status, previous history of GH, previous history of PE, family history of diabetes mellitus, family history of chronic hypertension (obtained from patient history alone), hemoglobin, random blood sugar, platelet count.

Clinical and laboratory data were recorded at each visit and during delivery in GH women. Participants were allowed to take rest for 10 to 15 min before blood pressure had been measured. Blood pressure measurements were taken while the women was seated using mercury sphygmomanometer apparatus which covers two third of the upper arm. The measurement was taken from right arm. The cuff was inflated at a rate of 2–3 mm Hg per second and deflated at a rate of 2–3 mm. First and fifth Korotkoff sounds were taken for systole and diastole respectively [10]. The following symptoms of PE were also obtained during each follow up: headache, right upper quadrant pain, epigastric pain, blurred vision, and abnormal body movement.

The following laboratory data at initial presentation and during follow up were additionally recorded in patients with GH: creatinine, aspartate amino transferase, urine protein. Women with GH were reviewed on average every 2 weeks until delivery, which is consistent with their regular follow up. Though follow-up until 12 weeks is required to confirm that women have not developed preeclampsia after birth, women were not followed for 12 weeks postpartum.

### Outcomes

The primary outcome was progression from GH to PE. Both maternal and perinatal outcomes were recorded. The maternal outcomes recorded were: number of maternal systemic complications (as mentioned above for PE under the heading” definitions”), antepartum hemorrhage (APH), mode of delivery. Additionally, the following perinatal outcomes were recorded: gestational age at delivery, birth weight, perinatal mortality, small for gestational age (SGA) rate, admission to neonatal intensive care unit (NICU).

### Statistical analysis

Incidence of GH was determined by dividing the entire number of exposures by the total number of deliveries during the study period. To determine the factors which might predict the development of PE, clinical and laboratory data at initial presentation were compared among women who developed PE and who did not. An a priori power analysis was undertaken to determine an adequate sample size for our primary outcome, progression from GH towards PE. We estimated that 112 participants would provide greater than 80 % power to identify difference between early and late gestational age at presentation in the primary outcome with odds ratio of 0.65 (95 % CI ,0.53–0.79) (Saudan et al. ) and two-sided significance level < 0.001 [4].

All statistical analyses were performed using the Statistical Package for Social Sciences for Windows 21.0 (SPSS Inc., Chicago, IL, USA). Values are expressed as mean ± SD. Continuous data (SD) were tested by analysis of variance and contingency tables were used for categorical data. Comparisons of participant characteristics were performed using a Pearson X^2^ test for categorical variables and two tailed student’s t test for continuous variables. P < 0.05 was considered significant. Binary logistic regression analysis was employed to assess the effects of the above clinical and laboratory data as predictors of progression from GH to PE. Then variables with P value less than or equal to 0.2 were fitted to multiple logistic regression. Moreover, clinically relevant variables were also fitted to the regression. Maternal age, gravidity, gestational age at diagnosis, history of abortion, family history of chronic hypertension, previous history of gestational hypertension, and hemoglobin were included in the model a priori. The predictive ability of the model was tested with Hosmer-Lemeshow goodness-of-fit test. Variables that were not significant were removed in a stepwise fashion. Finally variables with P-value ≤ 0.05 were considered as significant predictors of progression from GH to PE. After the purpose of the study has been informed, written informed consent was obtained from each study participants. Confidentiality of study participants was maintained.

## Results

During the one year study period; the total number of deliveries was 4002. Two hundred forty women with GH were seen giving an incidence of 6 % (95 CI 4.9–8.5). The mean age of the study participants was 27.15 years and SD of 5.33. Nearly half of the participants were nulliparous, mean gestational age at diagnosis of GH was 33 weeks. Family history of PE (1 %) and previous history of PE (2.1 %) was not common (Table 1).

**Table 1:** Characteristics of study participants at presentation in ACSH and MGH, North Ethiopia 2016/17

Variable	GH-GH	GH-PE	Total	P
N	199	41	240	
Age (years)	27.1 ± 2.1	29.6 ± 2.4		0.01
Nullipara	81 (40.7 %)	16 (39.0 %)	97 (40.4 %)	0.92
Gestational age at diagnosis of HDP (weeks)	36.7(± 4.0)	32.8 (± 4.0)	-	0.01
Family history of GH	4 (2.0 %)	1(2.4 %)	5 (2.1 %))	0.84
Family history of PE	2 (1.0 %)	-	2 (1 %)	0.52
Family history of chronic hypertension	17 (8.5 %)	3 (7.3 %)	20 (8.3 %)	0.01
Previous history of GH	3 (1.5 %)	19 (4.6 %)	22 (9.2 %)	< 0.0001
Previous of history PE	5 (2.5 %)	-	5 (2.1 %)	0.32
Pre-gestational diabetes	1 (0.5 %)	3 (7.3 %)	4 (1.7 %)	0.04
**Abortion**:				
First trimester	26 (13.1 %)	11 (26.9 %)	37 (15.4 %)	< 0.0001
Second trimester	-	7 (17.1 %)	7 (2.9 %)	< 0.0001
1st Trimester SBP mmHg	115(± 8)	118(± 7)	-	0.34
1st Trimester DBP mmHg	86(± 3)	79(± 5)	-	0.41
SBP at diagnosis mmHg	140(± 9)	145 (± 10)	-	0.36
DBP at diagnosis mmHg	91(± 7)	92(± 4)	-	0.37
Creatinine	0.6(± 0.2)	0.8 (± 1.3)	-	0.57
AST IU/L	25.8(± 12.0)	27.1(± 14.7)	-	0.51
Hemoglobin g/dl	13.5(± 1.6)	11.6(± 2.0)	-	< 0.0001
Platelet count	298.0(± 82.3)	284.3(± 210.3)	-	0.29

*APH *antepartum hemorrhage, *CS *cesarean section, *HDP *hypertensive disorders of pregnancy, *GH *gestational hypertension, *PE *preeclampsia, *SBP *systolic blood pressure, *DBP *diastolic blood pressure, *P *chi square for categorical variables and t test for continuous variables

Of the 240 women with GH, 41 (17.1 %, 95 CI 13.4–23.8) developed PE. The mean age of women who progressed to PE, 29.6 years, was older than those who remained with the diagnosis of GH, 27.1 years (*P* = 0.01). Those who presented with GH but developed PE presented at earlier gestational age than those who remained with GH (32.8 ± 4.0 weeks vs. 36.7 ± 4.0, *P* = 0.01). Their mean gestational age at progression to PE was 37.1 ± 3. Further, they had a lower level of hemoglobin, 11.6 ± 2.0 g/dl, (*P* < 0.0001) at presentation (Table 1).

Women who progressed from GH to PE had comparable SBP/DBP, 145 ± 10 mmHg/ 92 ± 4 mmHg, at diagnosis with those who remained with a diagnosis of GH until delivery,140 mmHg±/91 ± 7 mmHg (*P* = 0.36/P = 0.37).

There was no difference in terms of gestational age at delivery and birth weight between women who progressed from GH to PE and women who remained with a diagnosis of GH until delivery (*P* = 0.56 and *P* = 0.29).

The most commonly experienced symptoms were neurologic manifestations. Twenty-six women (63.4 %) among those who developed PE had one or more of the following neurologic symptoms; headache, blurring of vision and abnormal body movement. Among the neurologic symptoms, headache and blurred vision were the two most common presenting complaints. Of the 41 women who progressed to PE from an initial diagnosis of GH, 25 of them had these two neurologic manifestations. Other symptoms experienced were liver dysfunction 27 (65.9 %), severe hypertension 25(61 %), APH 10 (24.4 %), renal impairment 5(12.2 %) and thrombocytopenia 4 (9.8 %) (Table 2). Of the 41 women who progressed to PE, 37 women (90.24 %) had symptoms and clinical signs of PE at the time of progression. Twenty two women had symptoms and clinical signs of PE while having normal laboratory result. Only 4 (9.76 %) women had abnormal laboratory result as sole evidence of progression.

**Table 2:** Maternal and fetal outcomes of study participants, ACSH and MGH, North Ethiopia 2016/17. Values are given as N, mean (SD)

Variables	GH-GH ***N***=199	GH-PE ***N***=41	Total ***N***=240	***P***
**Maternal outcomes**				
Proteinuria	-	13 (31.7%)	13 (5.4%)	-
Renal impairment	-	5 (12.2%)	5 (2.1%)	-
Neurologic Disease				
Headache	-	20 (48.8%)	20 (8.3%)	-
Blurring of Vision	-	5 (12.2%)	5 (2.1%)	-
Abnormal body movement	-	1 (2.4%)	1 (0.4%)	-
Thrombocytopenia	-	4 (9.8%)	4 (1.7%)	-
Liver dysfunction				
Right upper quadrant pain	-	12 (29.3 %)	12 (5.0%)	-
Epigastric pain	-	6 (14.6%)	6 (2.5%)	-
Liver enzyme elevation	-	7 (17.1%)	7 (2.9%)	-
Sever hypertension	-	25 (61.0%)	25 (10.4%)	-
APH	4 (2.0%)	10 (24.4%)	14 (5.8%)	0.24
Mode of delivery				
Vaginal delivery	162 (81.4%)	29 (70.7%)	191 (79.6%)	0.58
Instrumental delivery	4 (2.0%)	-	4(1.7%)	0.36
CS delivery	33 (16.6%)	12(29.3 %)	45 (18.8%)	0.81
**Fetal outcomes**				
Gestational at delivery in weeks	38.6(2.4)	38.0 (2.0)	-	0.56
Birth weight in grams	3167.0(1236.2)	2917.1 (545.4)	-	0.29
1^st^ minute Apgar score >6	193 (97.0%)	40 (97.6%)	233 (97.1%)	0.98
1^st^ minute Apgar score 1-6	5 (2.5%)	1 (2.4%)	6 (2.5%)	0.98
Admission to NICU	26 (13.1%)	5 (12.2%)	31	0.06
Stillbirth	1	-	1	0.65

*APH* antepartum hemorrhage, *CS* cesarean section, *GH* gestational hypertension, *NICU* neonatal intensive care unit, *PE* preeclampsia, *P* Pearson X2 test for categorical variables and two tailed student’s t test for continuous variables

Univariate analysis revealed that maternal age, gestational age, history of abortion, family history of chronic hypertension, previous history of GH, and hemoglobin were factors associated with progression at 0.2 level of significance. Gravidity was included in the multivariate analysis because of its clinical significance.

However abortion, previous history of GH, and hemoglobin level remained significantly and independently associated with progression of GH towards PE in the multivariate analysis (Table 3).

**Table 3:** Univariate and multivariate logistic regression of factors associated with progression of GH towards PE among pregnant women in ACSH and MGH, 2016/ 2017 (*N* = 240)

Variables	Maternal outcome		OR (95 % CI)	
	**GH-GH** ***N*** = 199	**GH-PE** ***N*** = 41	**COR (95 %CI)**	**AOR (95 %CI)**
**Maternal age (year)**				
< 35	168(84.4 %)	31 (75.6 %)	1	
≥ 35	31(15.6 %)	10 (24.4 %)	1.74 (0.79–3.9)	
**Gestational age at diagnosis (week)**				
< 34	56 (28.1 %)	12 (29.3 %)	2.47 ( 0.76–5.35)	
≥ 34	143 (71.9 %)	29 (70.7 %)	1	
**Gravidity**				
1	81(40.7 %)	16 (39 %)	3.68 (0.89–4.56)	3.2 (1.32–5.64)
> 1	118(59.3 %)	25 (61 %)	1	1
**Abortion**				
Yes	173 (86.9 %)	19(46.3 %)	7.69(5.0-9.09)*	3.84(1.07-25.0)*
No	26 (13.1 %)	22(53.7 %)	1	1
**Previous history of GH**				
Yes	3 (1.5 %)	19 (46.3 %)	28.43 (2.92–49.12)*	26.76 (3.03–49.08)*
No	196 (98.5 %)	22 (53.7 %)	1	1
**Family history of chronic hypertension**				
Yes	17 (8.5 %)	3 (7.3 %)	2.13 (0.73–4.32)	
No	182 (91.5 %)	38 (92.7 %)	1	
**Hemoglobin (g/dl)**				
7-<11	13 (6.5 %)	22 (53.7 %)	13.71(3.96–22.40)*	13.1(1.40-17.27)*
>=11	186 (9.2 %)	19 (46.3 %)	1	1

*GH *gestational hypertension, *PE *preeclampsia, *COR *Crude odds ratio, *AOR *Adjusted odds ratio

*P-value < 0.05

Women who had previous history GH were 26 times more likely to develop PE in the consecutive pregnancy than women who had not had previous history GH (AOR = 26.76, 95 % CI: 3.03–49.08). Women who had abortion are 3.85 times more likely to develop PE in the consecutive pregnancy than their counterparts (AOR = 3.84. 95 % CI: 1.07-25.0).

Another significant association was found between hemoglobin levels and women who developed PE. Women with a hemoglobin level between 7 and 10 g/dl are 13times more likely to develop PE than women with hemoglobin level 11 g/dl and above (AOR = 13.1, 95 % CI: 1.40-17.27).

## Discussion

According to this study, the incidence of GH was 6 % (4.9–8.5) and the likelihood of progression from GH to PE was 17.1 % (13.4–23.8). This was more likely to occur in those who had history of abortion, previous history of GH, or low hemoglobin.

The overall incidence of GH in this study (6 %) is comparable with those previously reported studies which estimated 3–10 % [5, 11–13].

In our study, the rate of progression from GH to PE was 17.1 %. In agreement with a study by Saudan P. et al. which reported 15–25 % [4]. But lower than reported in another study by Barton et al. 46 %. It is good to mention that in the latter study the authors used urine dipstick + 1 as opposed to + 2 in our study to diagnose progression. Furthermore, when the authors used + 3 urine dipsticks, the rate of progression dropped to 9 %. The difference in urine dipstick cut off point used for the diagnosis of progression may explain the discrepancy in findings [14].

In this study women with previous history of GH were 26 times more likely to have PE in the subsequent pregnancy. This is much higher than a report from prospective study done in UK that showed women with previous GH were more likely to develop various obstetrics complications in subsequent pregnancies including 25 % risk of PE development [15]. The discrepancy may be explained due to set up difference. In our set up, urine dipstick is used to make a diagnosis of GH, potentially increasing its diagnosis.

Multiple studies have described that women with high hemoglobin level are likely to develop hypertensive disorders of pregnancy than those with normal level [16–19]. Elevated hemoglobin level can either be cause or effect of PE. There is limited intravascular expansion in women with PE leading to hemoconcentration. On the other end, the increased incidence of PE in pregnant women with elevated hemoglobin levels could be clarified by the toxic effects of haeme deposition on the vascular endothelium [20]. Interestingly, the present study found a significant association between low hemoglobin levels and progression towards PE. Women with a hemoglobin level between 7 and 10 were 13 times more likely to develop PE than women with hemoglobin level 11 and above. Another study by Abdulaziem et al. reported that severe anemia was risk factor for PE [21]. But the authors didn’t show association between mild/moderate anemia and PE. Moreover, the hemoglobin was determined after women presented with PE, posing difficulty in making cause and effect relationship. As other causes of anemia were not studied in the present study, it is difficult to determine if the anemia was the predictor.

This study found out that previous abortion is associated with risk of progression towards PE. Women who had an abortion were 3.85 times more likely to develop PE in the consecutive pregnancy than their counterpart. This is similar to published report by S Bhattacharya et al. that states women with history of abortion were 3.3 times more likely to suffer from PE compared to women with no previous history [22]. Other studies have showed that risk of PE to be related with the number of previous abortions, Mahdi S. et al. reported that the number of previous spontaneous abortions was found to be associated with PE. In this latter study, with each previous abortion the odds having PE in subsequent pregnancy was increased by 1.2 times [23]. Additionally Saudan P. et al. reported an association between history of abortion and chance of progression from GH-PE. However in a cohort study, Gunnarsdottir et al. found relationship between three previous abortions and PE not with one or two previous abortions [24]. In the present study we were not able to quantify number of previous abortions.

This study has found out that majority of the women (90.24 %) who progressed towards PE had signs and symptoms of PE (headache, blurring of vision, right upper quadrant pain, epigastric pain, abnormal body movement, and sever hypertension) at the diagnosis of progression. The remaining 9.76 % women had abnormal laboratory result as sole evidence of progression. Management of GH requires continued monitoring to detect transformation from this relatively mild disorder to the more severe disorder of PE [11]. However, either signs and symptoms or abnormal laboratory result is enough to make a diagnosis of progression [25]. This study has shown that majority of the women might have not required lab investigations to rule in or rule out PE. Thus, in low resource setting where laboratory investigations are absent signs and symptoms could be used to diagnose progression towards PE without resorting to laboratory investigations.

In our observation, comparison of perinatal outcome in between those who remained with GH alone till delivery and those who progressed to PE in terms of; birth weight, intrauterine growth restriction, admission to N-ICU, and 5th minute Apgar score and stillbirth was not statistically significantly different. This is similar to previous studies done in different centers which showed late onset PE had favorable perinatal outcome. Most common poor perinatal outcomes related to early onset PE include; fetal growth restriction, preterm delivery, poor Apgar score and stillbirth [26].

Study done in turkey by Riza M. et al. showed that incidences of small-for-gestational age, Apgar score < 7 at 5 min, and stillbirth were significantly higher in women with early onset PE compared to late onset PE [27]. Similarly, another cohort study by Wojtowicz et al. et al showed that women with late onset PE had better perinatal outcome as compared to early onset PE [28]. In our study the mean gestational age at progression towards PE was 37.1 ± 3 weeks. This late gestational age at progression might have led to comparable perinatal outcome among women who did and who did not progressed towards PE.

## Conclusions

The present study highlighted the incidence and rate of progression is similar to other studies done in low, middle, and high income countries.

Previous history of GH, anemia during pregnancy, previous second-trimester spontaneous abortion were significant predictors of progression towards PE.

The present study underlined that, in a resource-limited setting where predictive and diagnostic means are limited, the clinical picture of women should be taken into consideration for prediction and diagnosis of PE. Owing to the significant association found in-between rate of progression and low hemoglobin, further prospective research is required to identify if anemia was the cause or effect of PE.

### Limitation of the study

Definite diagnosis of GH is made retrospectively when the patient doesn’t develop PE and if blood pressure returns to normal by 12-week postpartum visit. In this study, subjects were followed until delivery only. Thus, hampering the true rate of progression of GH towards PE. Though BMI is reported in other studies as a risk factor for preeclampsia [29], we have not assessed it in this study; as most of the women did not recall their pre-pregnancy weight. Moreover, there are other clinical risk factors reported in other studies which were not assessed in this study. These clinical risk factors are hypercholesterolemia, Antiphospholipid syndrome, and thrombophilia [30, 31]. We were not able to assess these factors due to limitation in set up. Due to these limitations, we cannot state these risk factors are not important in the progression of PE. Although all cases seen during the study period were enrolled, smaller sample size has limited us from doing further analysis of variables with less frequent observations.

## Data Availability

The datasets generated and/or analysed during the current study are available from the corresponding author on reasonable request.

## Abbreviations

APH: Antepartum hemorrhage
**ACSH**: Ayder Comprehensive Specialized Hospital
CS: Cesarean section
DBP: Diastolic blood pressure
GH: Gestation hypertension
HDP: Hypertensive disorders of pregnancy
HTN: Hypertension
NICU: Neonatal intensive care unit
PE: Preeclampsia
SBP: Systolic blood pressure
